# Pyroptosis induced by enterovirus 71 and coxsackievirus B3 infection affects viral replication and host response

**DOI:** 10.1038/s41598-018-20958-1

**Published:** 2018-02-13

**Authors:** Yan Wang, Ying Qin, Tianying Wang, Yang Chen, Xiujuan Lang, Jia Zheng, Shuoyang Gao, Sijia Chen, Xiaoyan Zhong, Yusong Mu, Xiaoyu Wu, Fengming Zhang, Wenran Zhao, Zhaohua Zhong

**Affiliations:** 1 Department of Microbiology, Harbin Medical University, 157 Baojian Road, Harbin, 150081 China; 2 Department of Cell Biology, Harbin Medical University, 157 Baojian Road, Harbin, 150081 China; 3 Department of Cardiology, Harbin Medical University, 23 Youzheng Street, Harbin, 150001 China

## Abstract

Enterovirus 71 (EV71) is the primary causative pathogen of hand, foot, and mouth disease (HFMD), affecting children with severe neurological complications. Pyroptosis is a programmed cell death characterized by cell lysis and inflammatory response. Although proinflammatory response has been implicated to play important roles in EV71-caused diseases, the involvement of pyroptosis in the pathogenesis of EV71 is poorly defined. We show that EV71 infection induced caspase-1 activation. Responding to the activation of caspase-1, the expression and secretion of both IL-1β and IL-18 were increased in EV71-infected cells. The treatment of caspase-1 inhibitor markedly improved the systemic response of the EV71-infected mice. Importantly, caspase-1 inhibitor suppressed EV71 replication in mouse brains. Similarly, pyroptosis was activated by the infection of coxsackievirus B3 (CVB3), an important member of the *Enterovirus* genus. Caspase-1 activation and the increased expression of IL-18 and NLRP3 were demonstrated in HeLa cells infected with CVB3. Caspase-1 inhibitor also alleviated the overall conditions of virus-infected mice with markedly decreased replication of CVB3 and reduced expression of caspase-1. These results indicate that pyroptosis is involved in the pathogenesis of both EV71 and CVB3 infections, and the treatment of caspase-1 inhibitor is beneficial to the host response during enterovirus infection.

## Introduction

Enteroviruses is a group of small single-strand, positive-sensed RNA viruses in the *Enterovirus* genus of *Picornaviridae* family^1–3^. Some enteroviruses such as enterovirus 71 (EV71) and coxsackievirus B (CVB) can lead to severe diseases such as aseptic meningitis, brainstem encephalitis, myocarditis, and pancreatitis^4,5^. Enterovirus infections are especially common among children under five-year old, and it is one of the major causative pathogens that cause the outbreak of hand, foot, and mouth disease (HFMD), which affects millions of children in the Asian-Pacific region^6,7^. In 2015, there were up to two million HFMD patients with 129 deaths reported in China (http://www.nhfpc.gov.cn). CVBs are also important members of the *Enterovirus* genus^3,8,9^. The manifestations of CVB infection ranges from mild cold to severe myocarditis, pericarditis, meningitis, and pancreatitis^3^. Similar to EV71, CVB predominantly affects children and young adults^10^. In some cases, viral myocarditis caused by CVB infection can progress to cardiomyopathy, which may lead to heart failure and require heart transplantation^11^.

To date, it remains unclear about the pathogenesis of both EV71 and CVB infection. It is also unclear about the molecular mechanism of the host response to EV71 and CVB infection, although increased levels of cytokines^12^ and proteins involved in apoptosis, autophagy and ubiquitin-proteasome system have been implicated^13–17^.

Cell death is a key component of the host defense against microbial infection^18^. Pyroptosis is a unique form of programmed cell death which is characterized by cell lysis and proinflammatory feature^19^. Pyroptosis most frequently occurs during the infection of intracellular pathogens and it is likely to form part of the defense mechanisms of the host against infection^20^. In this process, cells recognize intracellular pathogens through a number of pattern-recognition receptors (PRRs) and form multi-protein complex, the inflammasome, which activates caspase-1^20^. The activation of caspase-1 converts the pro-interleukin (IL)-1β and pro-IL-18 to their mature forms which are released from the cells^21^. Studies in the recent years have identified gasdermin D (GSDMD) as the executioner of proptosis, which is the substrate of proinflammatory caspases (caspase-1, -11, -4, -5). The cleaved GSDMD forms non-selective pore in the plasma membrane, leading to pyroptosis^22,23^. The secretion of IL-1β and IL-18 and the release of cellular content due to cell lysis promote the recruitment of inflammatory cells, resulting in the activation of immune cells and the further production of cytokines^24^. However, excessive inflammation can have pathological consequences. Caspase-1 activation has been implicated in the pathogenesis of the diseases characterized by cell death and inflammation such as myocardial infarction^25^, ischemic brain injury^26^, neurodegenerative disease^27^, and intestinal inflammation^28^. However, the role of pyroptosis in the pathogenesis of enterovirus infection remains unknown.

This study aims to investigate the role of pyroptosis in the pathogenesis of EV71 and CVB3 infection. We started by measuring the activation caspase-1 and the secretion of IL-1β and IL-18 in cultured cells infected with EV71 or CVB3. We then studied the impact of pyroptosis on newborn mice during viral infection. Our results demonstrated that pyroptosis is involved in the pathogenesis of both EV71 and CVB3 infection.

## Results

### Caspase-1 is activated in HeLa cells infected with EV71

**Figure 1:**
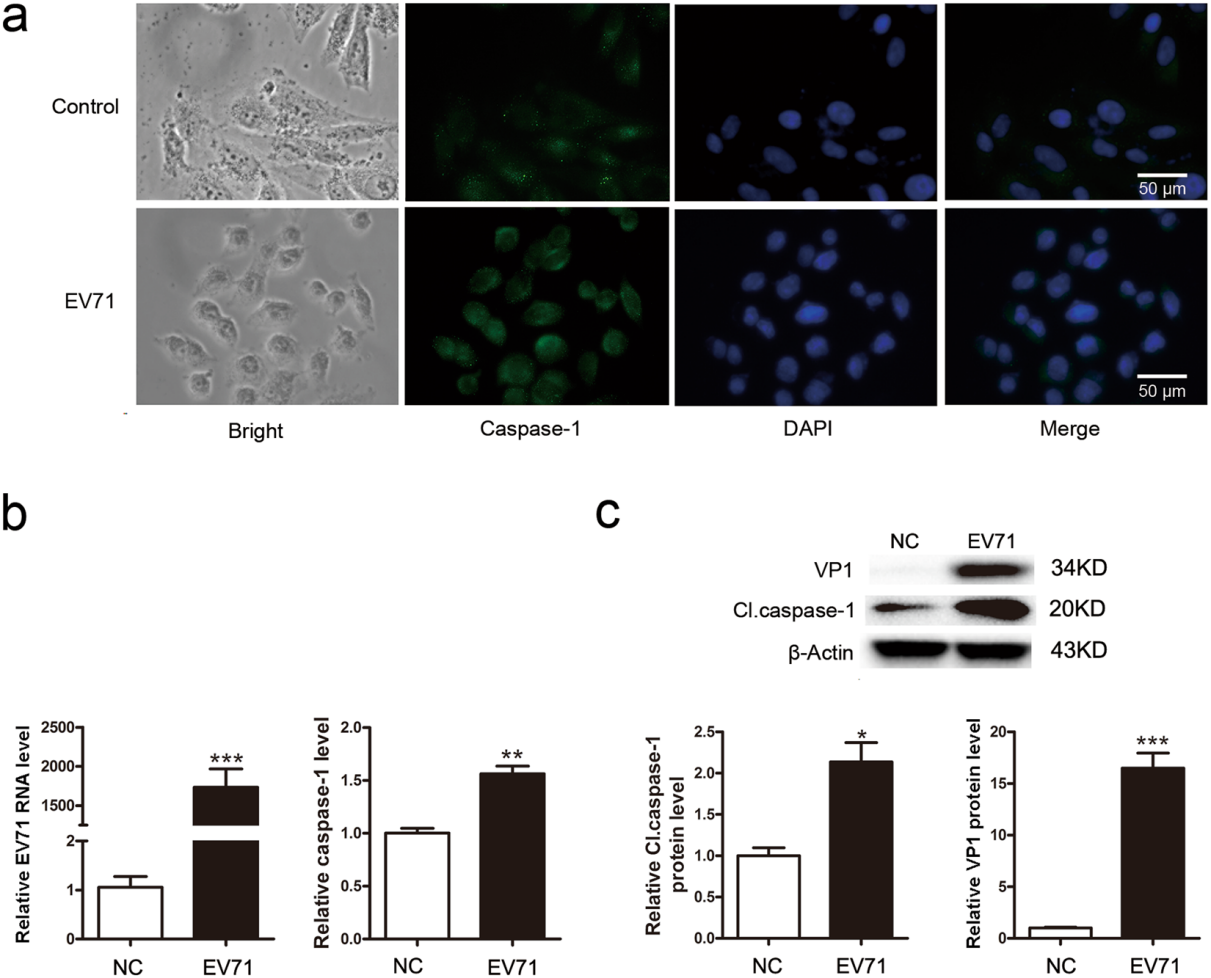
EV71 infection activated caspase-1 in HeLa cells. HeLa cells were infected with EV71 (MOI = 1) for 6 h and subjected to the examination of immunofluorescence microscopy (**a**) RT-qPCR (**b**) and Western blotting (**c**). Nuclei were stained with DAPI (**a**). *P < 0.05; **P < 0.01; ***P < 0.001. *n* = 4. Cl. caspase-1: cleaved caspase-1.

**Figure 2:**
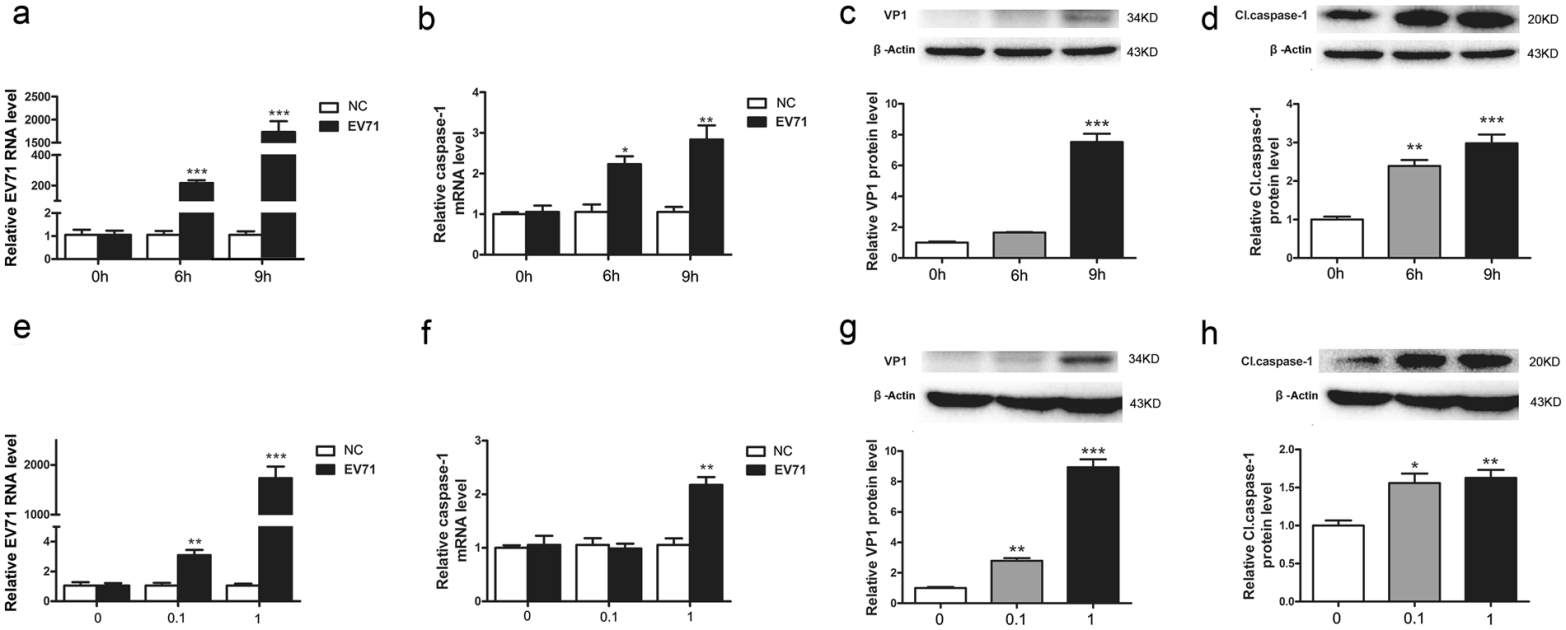
EV71 infection activated caspase-1 in time- and dose-dependent manner. HeLa cel ls were infected with EV71 (MOI = 1) and subjected to the examination of RT-qPCR (**a**,**b**) and Western blotting (**c**,**d**) at 0, 6, and 9 h of p.i. HeLa cells were infected with EV71 (MOI = 1 or MOI = 0.1) for 6 h and subjected to the analysis of RT-qPCR (**e**,**f**) and Western blotting (**g**,**h**). Statistical data were obtained by comparing the EV71-infected cells collected at 6 h or 9 h of p.i. with the cells collected at 0 h of p.i. (**c** and **d**), while cells infected with EV71 at various doses (MOI = 0.1 or 1) were compared with non-infected cells (**g** and **h**). *n* = 3. *P < 0.05; **P < 0.01; ***P < 0.001. Cl. caspase-1: cleaved caspase-1.

EV71 infection often induces extensive inflammatory response with abnormal cytokine and chemokine production^28–30^. However, if pyroptosis is stimulated by EV71 infection remains unknown. We started by measuring the activation of caspase-1. HeLa cells were infected with EV71 and the expression and activity of caspase-1 were determined. As shown in Fig. 1, the expression of caspase-1 was increased modestly but significantly both at mRNA and protein levels (Fig. 1a–c). Furthermore, the increased expression of caspase-1 was correlated with the replication of EV71 in a time- (Fig. 2a–d) and dose-dependent pattern (Fig. 2e–h), indicating that the increased level of caspase-1 was the result of the replication of EV71. In addition, caspase-1 was elevated in two to three folds at both mRNA and protein levels at 6 h post-infection (p.i.) (Fig. 2b and d), suggesting the activation of caspase-1 was a rapid cellular response to EV71 infection.

### EV71 infection increases the expression of IL-1β and IL-18 in HeLa cells

**Figure 3:**
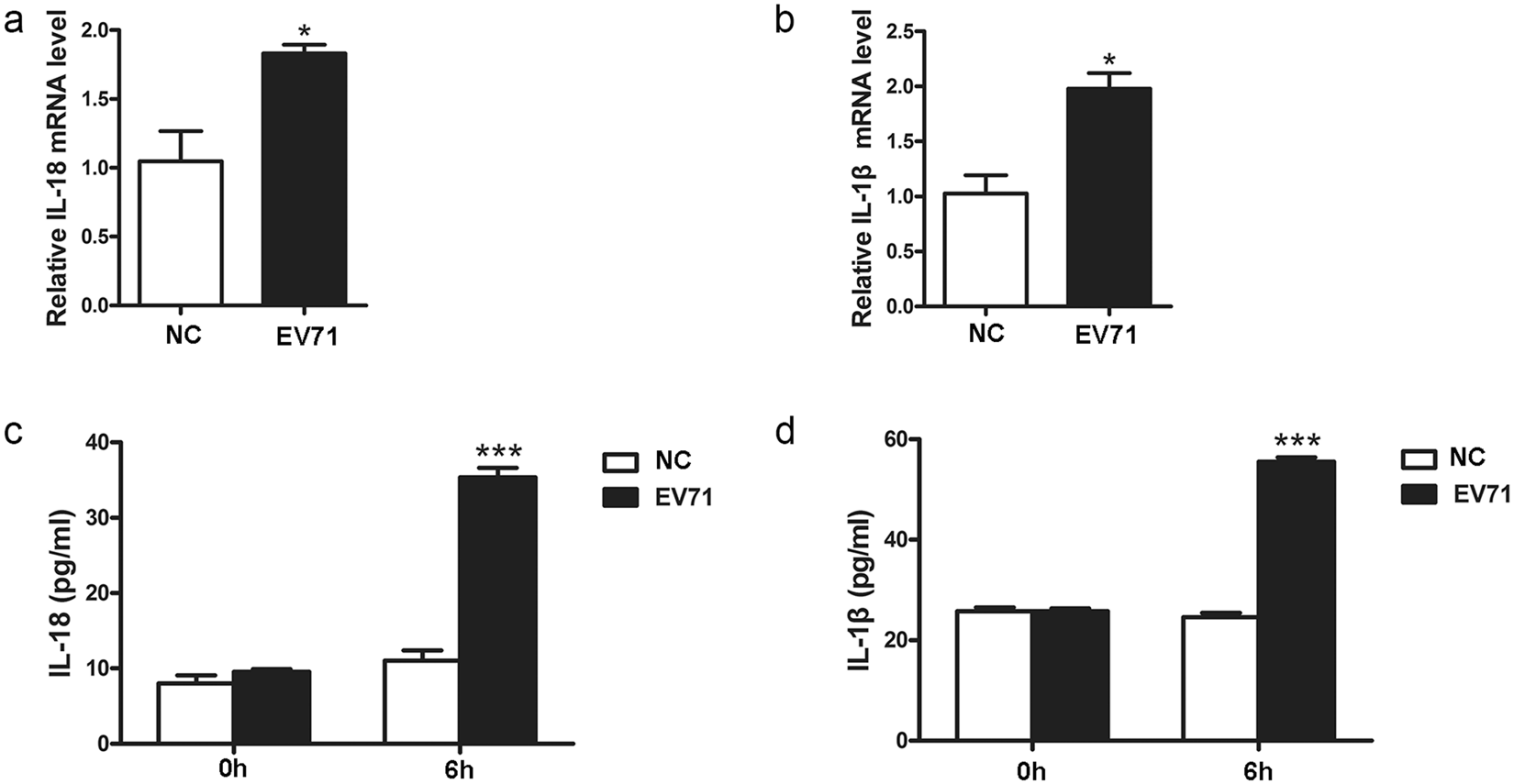
EV71 infection increased the expression of IL-1β and IL-18 in HeLa cells. HeLa cells were infected with EV71 (MOI = 1) for 6 h and subjected to RT-qPCR analysis (**a**,**b**). The cell culture supernatant was subjected to the examination of ELISA (**c**,**d**). *n* = 3. *P < 0.05; ***P < 0.001.

To demonstrate if the activation of caspase-1 is accompanied by the altered expression of IL-1β and IL-18, both cytokines were determined in HeLa cells infected with EV71. As shown in Fig. 3, IL-18 and IL-1β were increased in two to three folds at both mRNA and protein levels in the cells infected with EV71 (Fig. 3a–d). Both cytokines were markedly elevated at the early phase of EV71 replication (at 6 h p.i.) (Fig. 3c and d), corresponding to the rapid increase of caspase-1 in response to viral infection (Fig. 2a and d).

### Ac-YVAD-CMK supresses EV71 replication in mice

**Figure 4:**
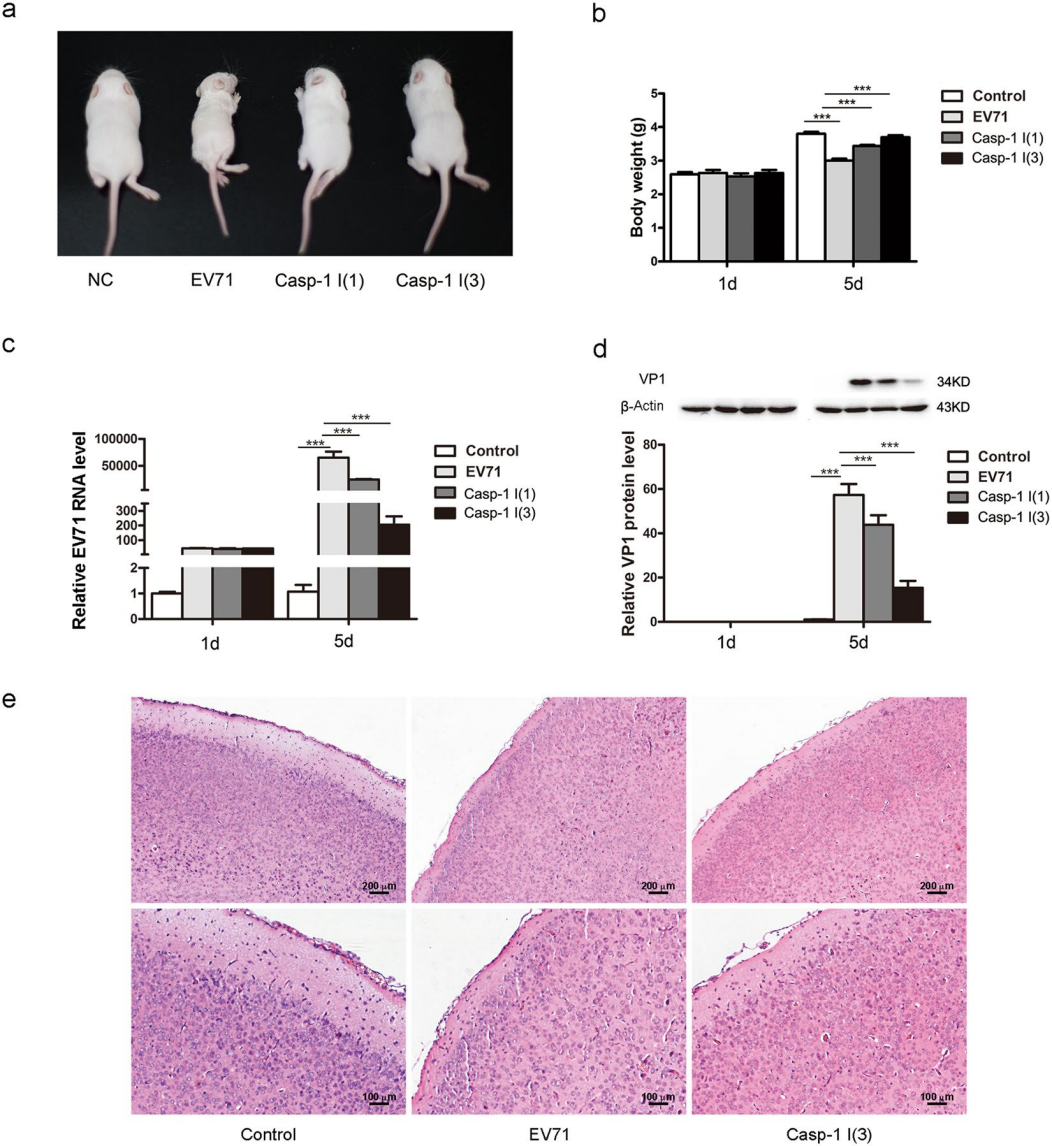
Ac-YVAD-CMK supressed EV71 replication in mice. Newborn Balb/c mice at the age three days after birth were inoculated with EV71 intraperitoneally. Mice were treated with Ac-YVAD-CMK intraperitoneally once at the same time of viral inoculation, or three time at 0, 2, 4 of p.i. (**a**). Body weight of the mice infected with EV71 was compared with that of the sham-infected mice (**b**). The body weights of the virus-infected mice treated with caspase-1 inhibitor were compared with that of the mice infected with EV71 (**b**). Total RNA and proteins were extracted from mouse brains and were subjected to the analysis of RT-qPCR (**c**) and Western blotting (**d**). Brain tissues of the mice at 5 d of p.i. were examined by immunohistochemistry (**e**). *n* = 3. ***P < 0.001. Casp-1 I (1): EV71-infected mice treated with caspase-1 inhibitor once. Casp-1 I (3): EV71-infected mice treated with caspase-1 inhibitor three times.

The above data indicate that pyroptosis is induced in the early stage of EV71 infection. We further evaluated the *in vivo* role of pyroptosis in response to EV71 infection in murine model. Neonatal Balb/c mice were infected with EV71 intraperitoneally. Mice were given caspase-1 inhibitor Ac-YVAD-CMK intraperitoneally once at the same time of viral infection or three times on day 0, 2, and 4 of p.i. Mice were sacrificed on various timepoints of p.i. and mouse brains were subjected to histological examinations. Viral replication was evaluated by RT-PCR and Western blotting. As shown in Fig. 4a, all mice survived on day 5 of p.i., but virus-infected mice showed wasting and hind limb paralysis (Fig. 4a and b). In contrast, mice treated with Ac-YVAD-CMK for three times showed significantly improved symptoms without limb paralysis (Fig. 4a and b), indicating that the administration of caspase-1 inhibitor alleviated the overall condition during EV71 infection. Tissue injury and mononuclear cell infiltration in the brains infected with EV71 were not apparent (Fig. 4e). Importantly, on day 5 of p.i., mice treated with Ac-YVAD-CMK showed significantly reduced viral replication (Fig. 4c and d), indicating that EV71 replication is suppressed by the treatment of Ac-YVAD-CMK.

### Ac-YVAD-CMK reduces the expression of caspase-1 in mouse brain infected with EV71

**Figure 5:**
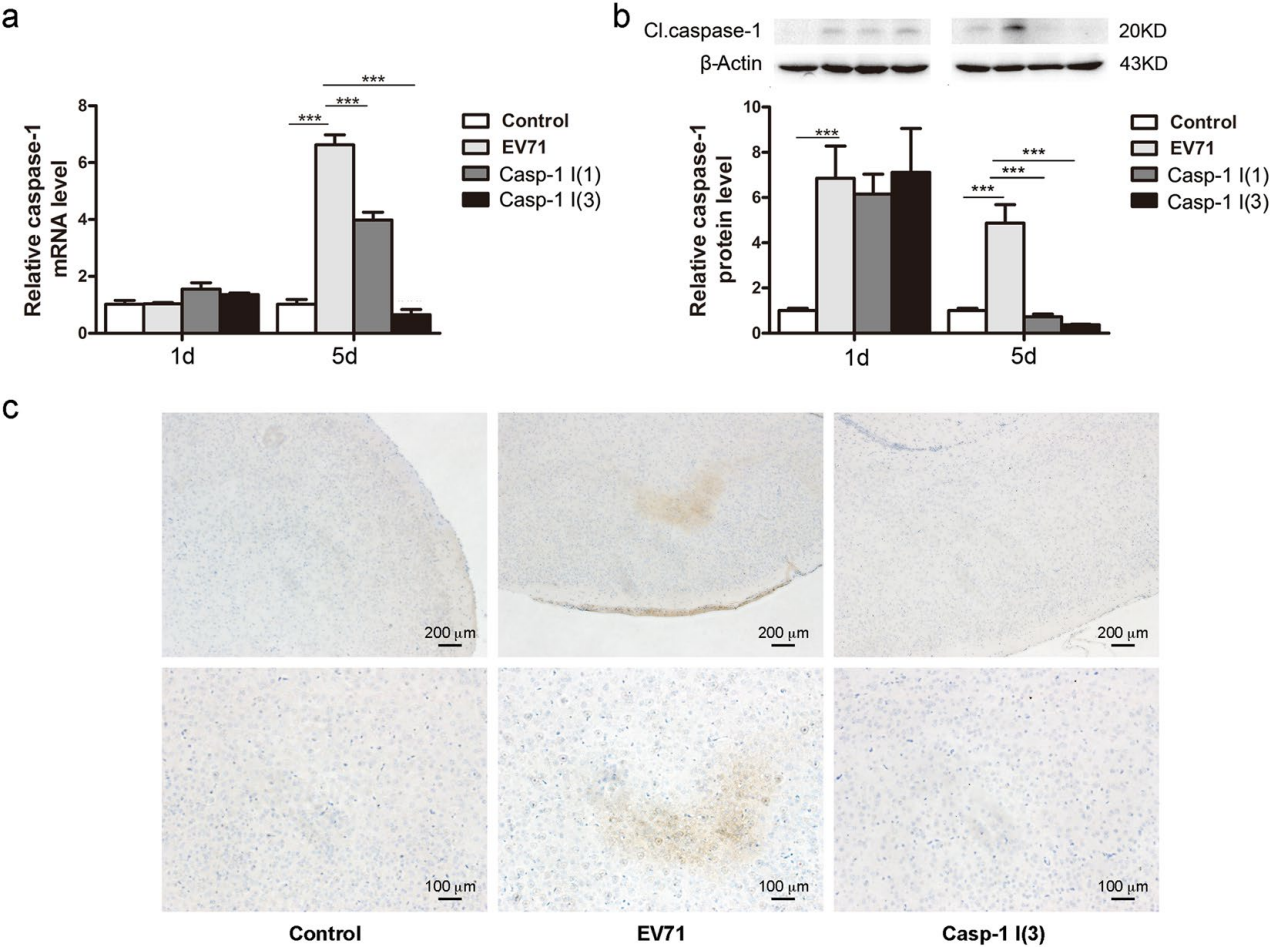
The reduced expression of caspase-1 in mouse brain infected with EV71 and treated with Ac-YVAD-CMK. Newborn Balb/c mice at the age three days after birth were infected with EV71 and treated with Ac-YVAD-CMK as described in Fig. 4. Total RNA and protein were extracted from mouse brains and subjected to the analysis of RT-qPCR (**a**) and Western blotting (**b**). The expression of caspase-1 was also determined by immunohistochemistry at 5 d of p.i. (**c**) *n* = 3. ***P < 0.001. Casp-1 I (1): EV71-infected mice treated with caspase-1 inhibitor once. Casp-1 I (3): EV71-infected mice treated with caspase-1 inhibitor three times.

To further probe the mechanism by which caspase-1 inhibitor Ac-YVAD-CMK improved the systematic symptom during EV71 infection, the neonatal mice were inoculated with EV71 and treated with Ac-YVAD-CMK as stated above. The expression of caspase-1 in mouse brains was evaluated. As shown in Fig. 5, on day 5 of p.i., the expression of caspase-1 was significantly decreased at both mRNA and protein levels, when mice treated with Ac-YVAD-CMK after viral infection for once (Fig. 5a and b) or three times (Fig. 5a to c) compared with that of the infected mice without Ac-YVAD-CMK treatment. These data imply that the improved symptom during EV71 infection is related with the suppressed pyroptosis and inflammation induced.

### CVB3 infection induces NLRP3-mediated pyroptosis

**Figure 6:**
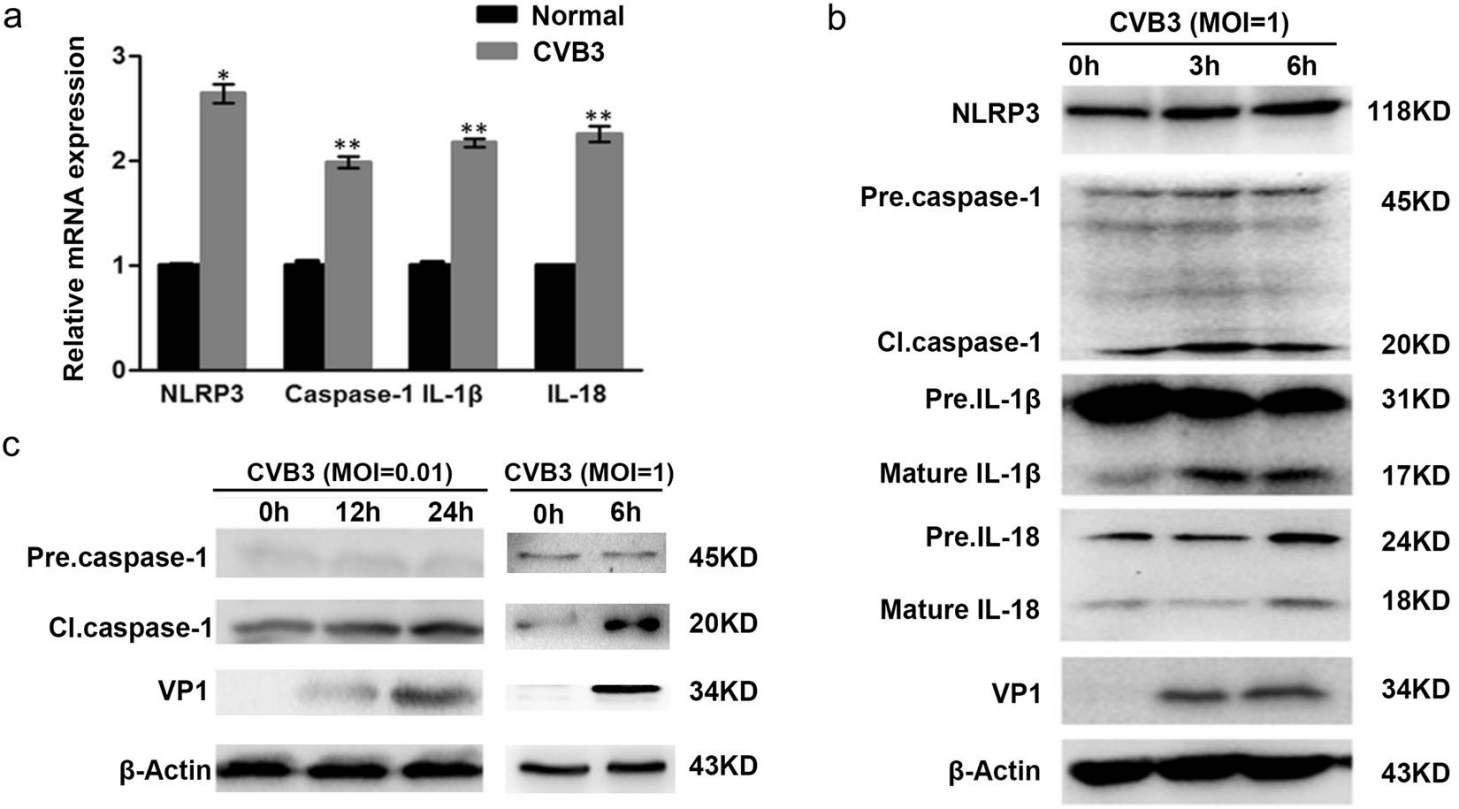
NLRP3-mediated pyroptosis was induced by CVB3 infection. HeLa cells were infected with CVB3 (MOI = 1) for 6 h and subjected to RT-qPCR analysis (**a**). Proteins were extracted from HeLa cells infected with CVB3 (MOI = 1) at various timepoints of p.i. and subjected to the analysis of Western blotting (**b**). HeLa cells were infected CVB3 (MOI = 0.01 or 1) and Western blot was performed at various timepoints of p.i. (**c**). *n* = 3. *P < 0.05; **P < 0.01. NLRP3: NOD-like receptor family pyrin domain containing 3. Pre. Caspase-1: the precursor of caspase-1. Pre. IL-1β: the precursor of IL-1β. Pre. IL-18: the precursor of IL-18.

Both CVB and enterovirus 71 are important members in the *Enterovirus* genus. Similar to EV71, CVB has also been demonstrated to infect central nervous system (CNS) and cause disorders such as encephalitis and aseptic meningitis^30–33^. We postulated that pyroptosis might also be involved in CVB infection. To this end, HeLa cells were infected with CVB3, and the expression of NOD-like receptor family pyrin domain containing 3 (NLRP3), caspase-1, IL-1β, and IL-18 was determined. As shown in Fig. 6, CVB3 infection significantly increased the expression of NLRP3, caspase-1, IL-1β, and IL-18 (Fig. 6a and b). The activation of caspase-1 occurred at 6 h of p.i., and it was correlated with CVB3 replication in a time- and dose-dependent manner (Fig. 6c). However, the precursor of caspase-1 remained unchanged (Fig. 6b and c). The active form of IL-1β increased as early as 3 h p.i., and the mature IL-18 was increased at 6 h p.i. (Fig. 6b). The protein level of NLRP3 was also elevated (Fig. 6b). These data collectively indicate that NLRP3-mediated pyroptosis was induced during the early stage of CVB3 infection.

### Ac-YVAD-CMK suppresses CVB3 replication and alleviates the inflammatory response in mice

**Figure 7:**
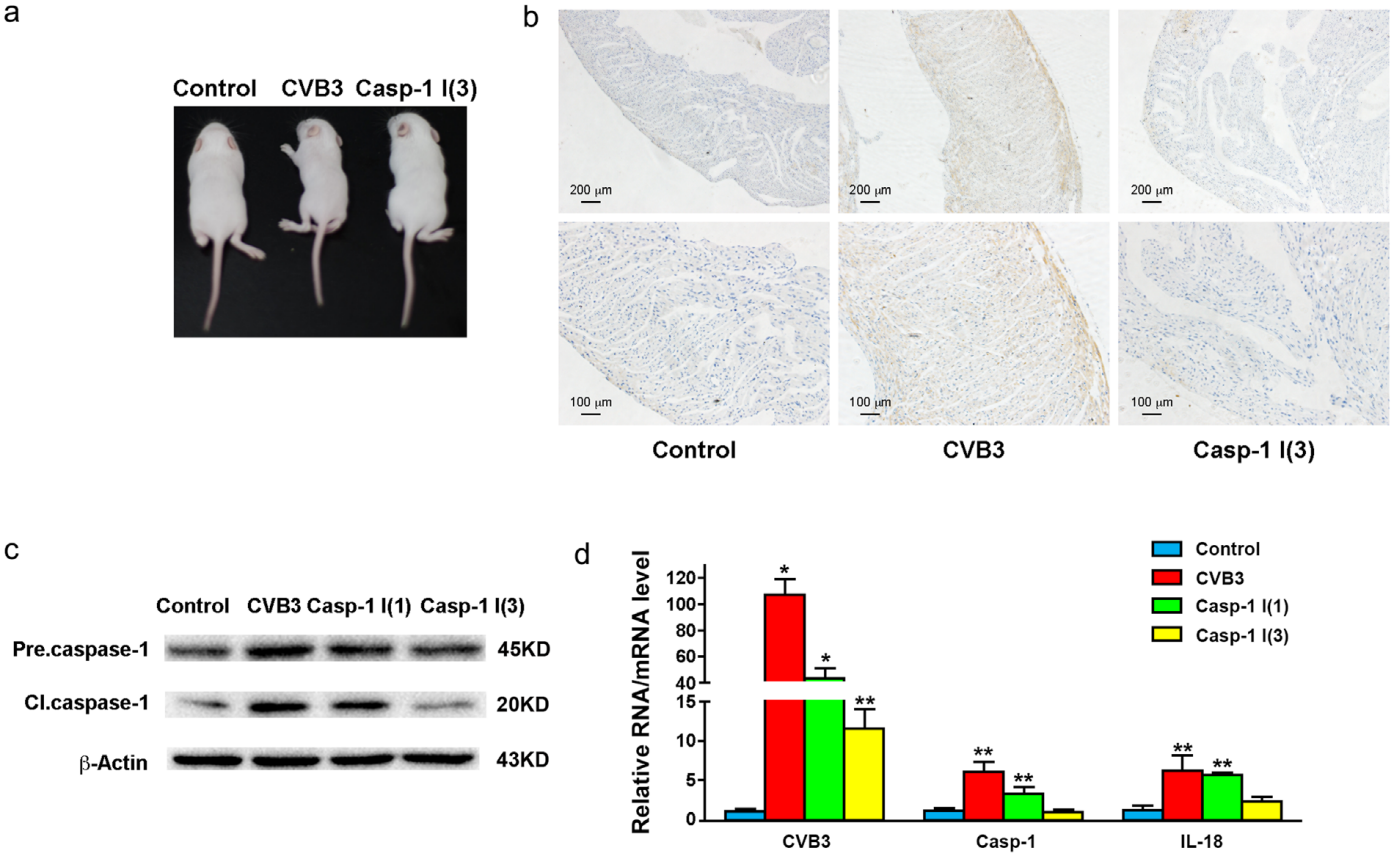
Ac-YVAD-CMK suppressed CVB3 replication and alleviated the inflammatory response in mice. Newborn Balb/c mice at the age three days after birth were infected with CVB3 intraperitoneally. Mice were treated with Ac-YVAD-CMK intraperitoneally once at the same time of viral inoculation, or three time at 0, 2, 4 of p.i. (**a**). Mouse cardiac muscles were examined by immunohistochemistry (**b**) at 5 d of p.i. Total proteins and RNAs were extracted form mouse myocardium and subjected to the analysis of Western blotting (**c**) and RT-qPCR (**d**). Statistical data were obtained by comparing the results between CVB3-infected mice with or without Ac-YVAD-CMK treatment and the control mice (**d**). *n* = 3. *P < 0.05; **P < 0.01. Casp-1: caspase-1. Casp-1 I (1): CVB3-infected mice treated with caspase-1 inhibitor once. Casp-1 I (3): CVB3-infected mice treated with caspase-1 inhibitor three times.

To evaluate the role of pyroptosis during CVB3 infection, newborn Balb/c mice at the age of 3 days were inoculated intraperitoneally with CVB3 and treated with caspase-1 inhibitor once at the same time when mice was infected with CVB3, or three times on day 0, 2, 4 of p.i. Mice were sacrificed on day 5 of p.i. and the expression of caspase-1 and IL-18 was determined. As shown in Fig. 7, all mice survived on day 5 of p.i. Mice infected with CVB3 showed significantly reduced body weight compared with the control mice (Fig. 7a, body weight not shown). In contrast, virus-infected mice treated with Ac-YVAD-CMK three times showed improved overall condition with body weight close to the control mice (Fig. 7a).

The increased expression and activation of caspase-1 were determined in cardiac muscles of the mice infected with CVB3 (Fig. 7b and c), while mice treated with Ac-YVAD-CMK showed significantly reduced expression and activation of caspase-1(Fig. 7b and c). The reduced expression of both caspase-1 and IL-18 in the myocardium of the mice treated with Ac-YVAD-CMK after CVB3 infection was also demonstrated at mRNA levels (Fig. 7d and e). Moreover, the treatment of Ac-YVAD-CMK resulted in the markedly reduced level of CVB3 RNA in the myocardium of mice (Fig. 7d). These data collectively demonstrated that inhibited pyroptosis suppressed viral replication and alleviated the inflammatory response during CVB3 infection.

### The increased secretion of IL-1β and IL-18 during the infection of CVB3 and EV71 depends on caspase-1

**Figure 8:**
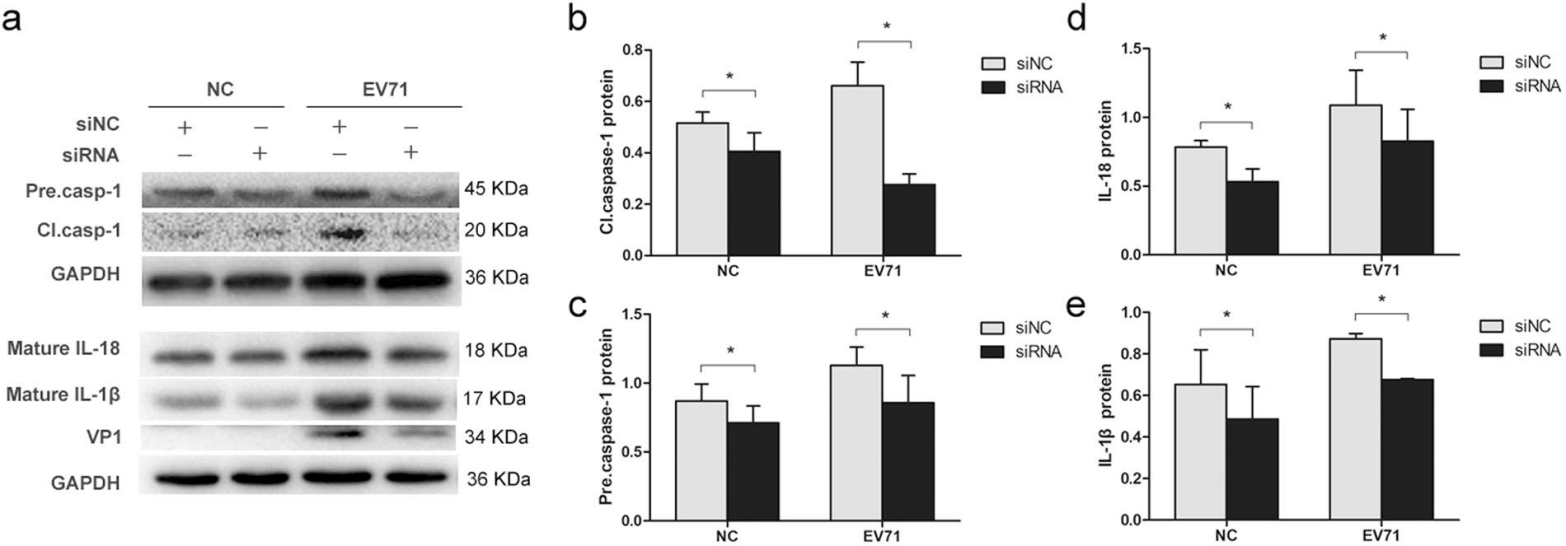
siRNA of caspase-1 prevents the increased secretion of IL-1β and IL-18 during the infection of EV71. HeLa cells cultured in 24-well plate were transfected with siRNA of the precursor of caspase-1 for 24 h and infected with EV71 at MOI = 0.1 for 12 h. Cellular proteins were extracted and subjected to Western blot analysis. Control cells were transfected with scramble siRNA (siNC) (**a**). Protein levels were presented as fold change relative to GAPDH (**b** to **e**). Experiment was repeated three times. Representative results were presented.

**Figure 9:**
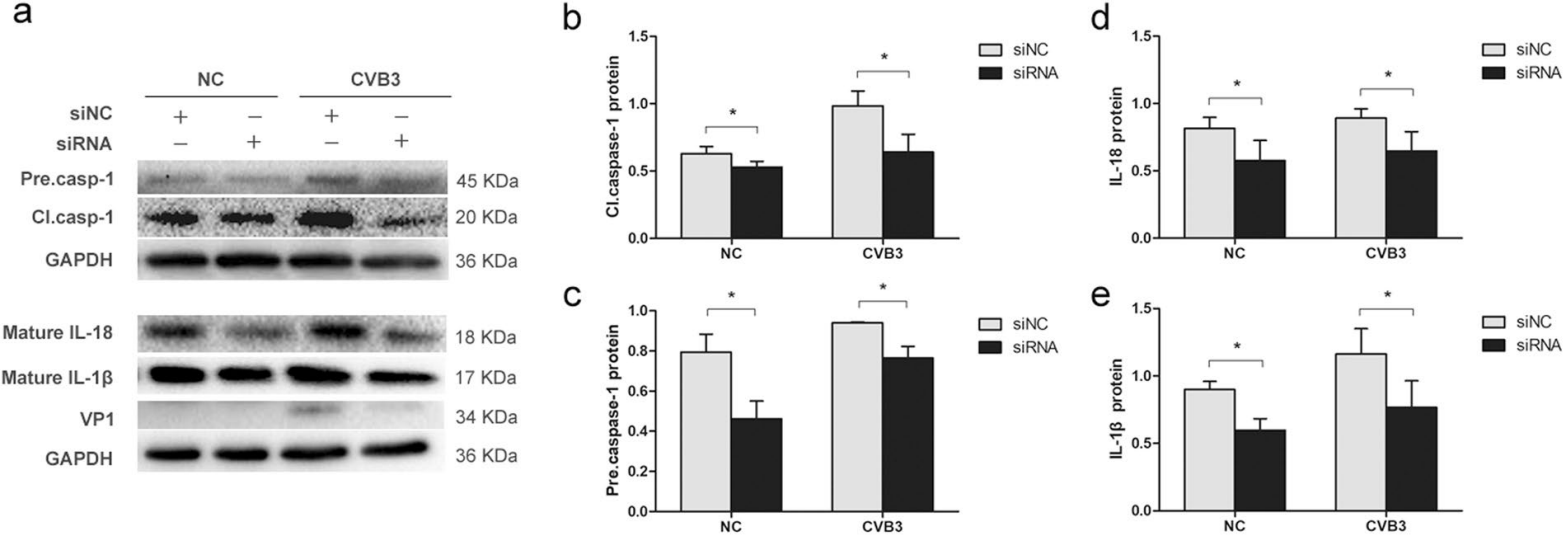
siRNA of caspase-1 prevents the increased secretion of IL-1β and IL-18 during the infection of CVB3. HeLa cells cultured in 24-well plate were transfected with siRNA of the precursor of caspase-1 for 24 h and infected with CVB3 at MOI = 0.1 for 12 h. Cellular proteins were extracted and subjected to Western blot analysis. Control cells were transfected with scramble siRNA (siNC) (**a**). Protein levels were presented as fold change relative to GAPDH (**b** to **e**). Experiment was repeated three times. Representative results were presented.

The data above show that the infection of both CVB3 and EV71 elicited pyroptosis. Since caspase-1 inhibitor AC-YVAD-CMK also shows inhibitory activity against caspase-4 and caspase-5, which also belong to inflammatory caspases, we asked the question whether caspase-1 plays a prominent role in pyroptosis during the infection of CVB3 and EV71. To this end, the expression of caspase-1 was knocked down by the siRNA of caspase-1 precursor (Santa Cruz) in the cells infected with CVB3 or EV71, and the levels of the mature IL-1β and IL-18 were determined by Western blotting. As shown in Fig. 8, when the expression of caspase-1 was inhibited by caspase-1 siRNA (Figs 8a–c and 9a–c), the infection of CVB3 or EV71 failed to elicit the elevated maturation of both IL-1β and IL-18 (Figs 8a,d,e and 9a,d,e), indicating that the maturation of both cytokines depends on caspase-1 during viral infection.

## Discussion

Diseases caused by the infection of EV71 and CVB are common among children and young adults^34,35^. A series of severe complications are related to enterovirus infection such as meningitis, encephalitis, acute flaccid paralysis, and myocarditis^5,36^. However, the pathogenesis of enterovirus infection is still not clearly understood. Evidence has shown that the inflammatory response of the host contributes to the pathogenesis of enterovirus infection^32^. Pyroptosis is a form of programmed cell death which leads to cell lysis and inflammation^37^. Pyroptosis plays an important role in host defence against microbial infection by removing the intracellular replication niche of the pathogen^38^. In this study, we explored the role of pyroptosis during the infection of EV71 and CVB3 both *in vitro* and *in vivo*.

Inflammatory response is crucial in fighting viral infection and establishing immunity. However, systemic inflammation can be harmful and it has been implicated in the pathogenesis of enterovirus infection. Elevated level of IL-1β have been demonstrated in the serum of both EV71-infected patient with encephalitis and mouse models^39^. The increased serum level of IL-1β, together with IL-6 and tumour necrosis factor (TNF)-alpha was associated with the severity of EV71 infection^40^. However, if pyroptosis is involved in the pathogenesis of EV71 infection remains unclear. Our *in vitro* study demonstrated that EV71 infection markedly increased the expression and activation of caspase-1 in HeLa cells as early as 6 h of p.i. (Figs 1 and 2). The increased expression and secretion of IL-1β and IL18 were also demonstrated in the cells infected by EV71 (Fig. 3). These results indicate that EV71 infection elicited pyroptosis. In agreement with our findings, study of Wang *et al*. demonstrated the activation of NLRP3 inflammasome and the secretion of IL-1β in human monocytic cell line THP-1 infected by EV71^41^. The activation of NLRP3 inflammasome was also found in the infection of encephalomyocarditis virus^42^. The reported data and our results collectively suggest that pyroptosis is induced at the early stage of EV71 infection.

Our further study indicates that pyroptosis contributes to the pathogenesis of enterovirus infection. First, we explored the role of pyroptosis in the neurological pathology of EV71 infection. In agreement with the previous report^43^, no significant histopathological change such as the infiltration of mononuclear cells in the brains infected with EV71 was observed (Fig. 4e), possibly due to the acute phase of viral infection. However, compared with the uninfected mice, EV71 infection led to limb paralysis and wasting of the neonatal mice (Fig. 4a and b). In contrast, the disease manifestations were not observed in the EV71-infected mice treated with caspase-1 inhibitor. Importantly, caspase-1 inhibitor Ac-YVAD-CMK markedly reduced the replication of EV71 in mouse brains (Fig. 4c and d). These findings imply that the treatment of caspase-1 inhibitor suppressed viral replication and improved the symptom during EV71 infection. Our results also imply that pyroptosis and the ensuing inflammation are harmful to the host during EV71 infection. Similarly, Hoegen, T. *et al*. found that NLRP3 knockout mice infected with streptococcus pneumoniae presented with less severe disease manifestation and brain inflammation^44^. However, study by Rajan, J. V., *et al*. indicated that pyroptosis or caspase-1 related inflammation may not determine the outcome of viral infection, since there was no difference for the survival rate between wild type mice and caspase-1 knockout mice infected with encephalomyocarditis virus (EMCV), another RNA virus in *Picornaviridae* family^45^. Still, further study is needed to elucidate the interplay between EV71 infection and pyroptosis.

Pyroptosis results in cell lysis and the secretion of proinflammatory cytokines, IL-1β and IL-18^37^. IL-1β has been proposed to be involved in the pathogenesis of EV71 infection, especially in the cases with neurological complications^46^. In the cerebrospinal fluid, the levels of IL-1β, IL-6, IL-8, and interferon (IFN)-γ were elevated^46,47^. Resident cells in the CNS such as neurons, astrocytes, and microglia responded to EV71 infection by releasing IL-1 β and TNF-α^48^. However, data are not available concerning the alteration of IL-18 during EV71 infection. In this study, dramatically elevated expression of caspase-1, IL-1 β, and IL-18 was observed in HeLa cells infected with EV71 (Fig. 3), and a marked increase in the expression of caspase-1 in mouse brains during EV71 infection was also demonstrated (Fig. 5). These data suggest that pyroptosis and the ensuring inflammation elicited by the proinflammatory cytokines play important roles in the pathology of CNS during EV71 infection.

Pyroptosis operates to remove the intracellular replication niche of pathogens and trigger inflammation^24,49–52^. However, for enterovirus infection involved in CNS and myocardium, inflammation is often more harmful than beneficial^39,40,53,54^. Both IL-1β and IL-18 have been implicated in the pathogenesis of neuroinflammation. Clinical investigation showed elevated plasma levels of IL-1β, together with other proinflammatory cytokines such IL-6 and TNF-α in patients with severe complications such as encephalitis and pulmonary oedema^40^. Brain is sensitive to IL-1β and IL-18 signaling at both a systemic and local levels, because multiple cell types in the CNS express the receptors of these cytokines^55–58^. Viruses, on the other hand, may evade or subvert cellular inflammatory response. Study has shown that EV71 could activate NLRP3-inflammasomes and at the same time inhibit the secretion of IL-1β by cleaving NLRP-3 with viral proteases 2A and 3C. Thus, the involvement of pyroptosis in the pathogenesis of EV71 infection could be more complicated than we expected^41^.

We also investigated the role of pyroptosis during CVB3 infection. *In vitro* study demonstrated that the activation and maturation of caspase-1, IL-1β, and IL-18 appeared as early as 3 or 6 h of p.i. (Fig. 6). The expression of NLRP3 was also increased. These data indicate that pyroptosis is the one of early cellular responses toward CVB3 infection. Caspase-1 was activated in the myocardium of mice on day 5 of CVB3 infection (Fig. 7b to d), which also promoted the expression of precursor of caspase-1 (Fig. 7c). The treatment of caspase-1 inhibitor dramatically reduced the expression of caspase-1 and IL-18 in myocardium (Fig. 7b to d) and alleviated the overall manifestations of the mice infected with CVB3 (Fig. 7a). More importantly, the treatment of caspase-1 inhibitor suppressed the CVB3 replication in mouse myocardium (Fig. 7d). Since caspase-1 inhibitor Ac-YVAD-CMK also shows inhibitory activity against other inflammatory caspases such as caspase-4 and caspase-5, we analysed the levels of the secreted IL-1β and IL-18 in the culture media of the cells infected with CVB3 or EV71 when caspase-1 was knocked down by siRNA. We demonstrated that viral infection failed to induce the elevation of the mature pro-inflammatory cytokines when the expression of caspase-1 was inhibited (Figs 8–9), indicating that caspase-1 plays an essential role in the pyroptosis elicited by the infection of CVB3 or EV71. These data indicate that Ac-YVAD-CMK has anti-CVB3 effect, and pyroptosis is harmful to myocardium, at least in the acute phase of CVB3 infection. Similarly, study by Wang, Y., *et al*. showed that the inhibition of NLRP3-inflammasome alleviated myocarditis and improved the cardiac function of the mice infected with CVB3^59^. The elevated expression of caspase-1 during EV71 and CVB infection might be the results of the activated NF-κB^60,61^, which regulates the expression of caspase-1^62^. Furthermore, IL-1β may promotes the expression of caspase-1 through IL-1 receptor/NF-κB pathway^63^, leading to a positive feedback in which the activation of caspase-1 promotes the expression of caspase-1. To reveal the antiviral mechanism of caspase-1 inhibitor, the impact of Ac-YVAD-CMK on the apoptosis of the virus-infected cells was analysed, but no significant anti-apoptotic effect was observed (supplementary material). Therefore, the antiviral mechanism of Ac-YVAD-CMK remains to be further investigated.

Although the interplay between pyroptosis and enterovirus infection needs further investigation, we postulate here that pyroptosis induced by enterovirus infection may not lead to the establishment of antiviral status to eliminate the intracellular EV71 or CVB3, since both viruses have been demonstrated to evade antiviral immunity by cleaving the proteins involved in innate immunity such as mitochondrial antiviral signalling protein (MAVS), melanoma-differentiation-associated (MDA5), and retinoic acid-inducible gene I (RIG-I) with viral proteases^64–66^. Instead, pyroptosis may promote the release and spread of viruses, leading to the infection of more cells. Therefore, the overall effect of pyroptosis is detrimental, at least in the acute phase of EV71 and CVB3 infection.

In summary, this study shows that pyroptosis is induced at the early stage of EV71 and CVB3 infection with the activation of caspse-1 and the secretion of IL-1 β and IL-18. The suppressed pyroptosis alleviated the inflammatory response of the virus-infected mice and reduced the replication of viruses in both CNS and myocardium. Our findings suggest that pyroptosis plays a critical role in the pathogenesis of EV71 and CVB3.

## Materials and Methods

### Ethics statement

Animal experiments were conducted in accordance with the guidelines of the Laboratory Animal Centre of Harbin Medical University. The protocols of animal experiment were approved by the Ethics Committee of Harbin Medical University. Mice were euthanized by 100% CO_2_ inhalation for 5 minutes followed by cervical dislocation to minimize the animals suffering after the completion of the experimental protocol. To perform virus infection, mice were placed in the anesthetic inhalator chamber containing isoflurane (Initial phase: 5%, Maintained phase: 1.5%~2.5%) for 1 minute before the intraperitoneal inoculation of viruses.

### Cell culture and virus

HeLa cells were maintained in the Department of Microbiology, Harbin Medical University (Harbin, China). Cells were grown in DMEM (Invitrogen, Shanghai, China) medium supplemented with 10% fetal bovine serum (FBS, Biological Industries, Israel), 100 U/ml penicillin, and 100 μg/ml streptomycin at 37 °C with 5% CO_2_. EV71 BrCr strain was provide by Center of Disease Control of Heilongjiang Province (Harbin, China). CVB3 Nancy strain was provided by the Center for Endemic Disease Control of China. Virus was propagated in HeLa cells and titrated by measuring the 50% tissue culture infective dose (TCID_50_).

### Viral infection of cell lines and mice

Exponentially growing cells were infected with the EV71 or CVB3 for 1 hour. Cell monolayers were then washed twice with PBS and incubated in complete culture medium. Cells or supernatants were harvested at various time points of p.i. and processed for further analyses. Newborn Balb/c mice were purchased from Shanghai Slack Laboratory Animal Cooperation (Shanghai, China). Mice were bred and maintained in the Laboratory Animal Center of Harbin Medial University. Each mouse at the age of three day after birth was inoculated intraperitoneally with 5 × 10^6^ TCID_50_ of EV71 or 1 × 10^6^ TCID_50_ of CVB3 in 20 μL cell lysate. 100 μM Ac-YVAD-CMK (Cayman Chemical) was given intraperitoneally to each mouse once on day 0 of p.i., or three times on day 0, 2, and 4 of p.i. Mice were sacrificed on various timepoints of p.i. and subjected to further analyses.

### Real-time quantitative PCR

HeLa Cells were infected with EV71 (MOI = 1) for 6 h. Total RNA was extracted using TRizol (Invitrogen, Carlsbad, CA) following the instructions of the provider. Reverse transcription was performed using PrimeScript reverse transcription kit (TaKaRa, Shiga, Japan). cDNA was obtained and real-time quantitative PCR (RT-qPCR) was performed using SYBR Premix EX Taq II (TaKaRa) and specific primers (Table S1) in a total reaction volume of 20 μL in Light Cycler 96 (Roche). Amplification of cDNA was performed for 40 cycles through denaturation at 98 °C for 5 s, primer annealing at 55 °C for 20 s, and extension at 72 °C for 15 s. Cycle threshold (Ct) value was calculated and 2^−ΔΔCt^ method was used to measure the levels of intracellular mRNA^67^. RNA levels were normalized to GAPDH RNA and presented as fold change. PCR Primers are listed in the supplementary material.

### Western blot

Cells were washed with cold PBS and treated with RIPA buffer (Thermo, Rockford, IL) containing protease inhibitor cocktail (Roche) and 1% phenylmethylsulfonyl fluoride PMSF (Beyotime, Shanghai, China). Proteins were quantified by Bradford assay and separated in 12% SDS-PAGE prior to transferring onto polyvinylidene difluoride (PVDF) membrane. Protein bands were visualized by enhanced chemiluminescence technique using SuperSignal West Pico chemiluminescent substrate (Thermo, USA). Densitometric quantification was performed using ImageJ v1.48 and the relative band intensity for each protein of interest was normalized against β-actin. The primary antibodies used in this study included antibodies against β-actin (Proteintech, Wuhan, China), caspase-1 (Cell Signaling), EV71 VP1(Abnova), IL-1β (Proteintech), IL-18 (Proteintech), and NLRP3 (Biovector, Shanghai, China). The goat anti-rabbit or anti-mouse horse radish peroxidase (HRP)-labeled antibodies were obtained from Proteintech.

### ELISA

The supernatant of cell culture was collected and IL-1β and IL-18 were determined by the enzyme-linked immunosorbent assay (ELISA) following  the instructions of the provider (Boster, Wuhan, China). After color development, the optical density at 450 nm was determined by microplate reader Epoch 2 (BioTek).

### Immunofluorescence

Cells growing on coverslips were rinsed with PBS three times for 5 min each and then fixed with 4% paraformaldehyde for 30 min. Cells were permeabilized with 0.1% Triton-100 for 15 min followed by three washes with PBS. The coverslips were blocked with 1% BSA in PBS for 30 min at 37 °C and then incubated with primary antibody at a dilution of 1:100 at 4 °C overnight. Cells were incubated with FITC-conjugated anti-rabbit IgG (H + L) antibodies. After three washes, the cells were incubated with 1 μg/ml DAPI in PBS for 5 mins. The coverslips were observed under Axiovert 200 (Zeiss) fluorescence microscope.

### Histological observation by light microscopy

Brain tissues of the mice were collected and subjected to histological observation by light microscopy. Tissues were fixed, dehydrated through gradient ethanol, and treated with xylene. Tissue samples were embedded in paraffin. 4 μm-thick paraffin-embedded tissue sections were placed onto siliconized slides and stained with hematoxylin and eosin. The stained sections were viewed by two histologists in the Department of Pathology of Harbin Medical University.

### Immunohistochemistry

Tissue sections were dewaxed, dehydrated. Antigen was retrieved in microwave in sodium citrate solution. Sections were then incubated with 3% H_2_O_2_ for 15 min and blocked with mouse immunoglobulin G blocking reagent (Vector Laboratories, Burlingame, CA) for 1 h. Sections were incubated with anti-caspase-1 (Cell Signaling) or anti-IL-18 (Proteintech) polyclonal antibodies at a dilution of 1:200 for 30 minutes, and then incubated with goat anti-rabbit HRP-labeled antibodies for 30 min. Sections were observed with a light microscope after counterstaining. Control sections were incubated with PBS instead of the specific primary antibodies.

### Statistical analysis

Student’s *t*-test was used to evaluate the data using GraphPad Prism software. The data shown are the mean ± SEM obtained from three independent experiments. The *P* value ≤ 0.05 was considered statistically significant.

## Electronic supplementary material

Supplementary information 
