# Supplementary material for: Pyroptosis induced by enterovirus 71 and coxsackievirus B3 infection affects viral replication and host response
Source: Sci Rep. 2018 Feb 13;8:2887. doi: 10.1038/s41598-018-20958-1 (PMC5811489; doi:10.1038/s41598-018-20958-1)
Supplement: Supplementary file 1 — Supplementary information [file 41598_2018_20958_MOESM1_ESM.pdf]

Pyroptosis induced by enterovirus 71 and coxsackievirus B3 infection affects viral replication and host response

Yan Wang[1], Ying Qin[1], Tianying Wang[1], Yang Chen[1], Xiujuan Lang[1], Jia Zheng[1], Shuoyang Gao[1], Sijia Chen[1], Xiaoyan Zhong[1], Yusong Mu[1], Xiaoyu Wu[3], Fengming Zhang[1], Wenran Zhao[2*], Zhaohua Zhong[1*]

[1]Department of Microbiology, Harbin Medical University, 157 Baojian Road, Harbin 150081, China

[2]Department of Cell Biology, Harbin Medical University, 157 Baojian Road, Harbin 150081, China

[3]Department of Cardiology, Harbin Medical University, 23 Youzheng Street, Harbin 150001, China

E-mails of the authors:

wangyan@hrbum.edu.cn (Y.W.)

wangty0929@163.com (T.W.)

qinyinggaofeng@163.com (Y.Q.)

cy_hmu@126.com (Y.C.)

langxiujuan11@163.com (X.L.)

sijiachen@163.com (S. C.)

littlerock712@163.com (X.Z.)

xiaoyu_wu2006@163.com (X.W.)

7765454@163.com (S.G.)

13920899969@126.com (J.Z)

604091916@qq.com (Y.M.)

fengmingzhaong@ems.hrbmu.edu.cn (F.Z.)

zhaowenran2002@aliyun.com; or zhaowr@ems.hrbmu.edu.cn (W.Z.)

zhonghmu@hrbmu.edu.cn, zhonghum@126.com (Z.Z.)

*Correspondence should be addressed to:

Zhaohua Zhong: zhonghmu@hrbmu.edu.cn, or zhonghum@126.com

Wenran Zhao: zhaowr@hrbmu.edu.cn, or zhaowenran2002@aliyun.com

Supplementary table 1. Primer sequences used for RT-qPCR.

Primer	Sequence (5′→3′)
m-NLRP3-F	TTCAATGGCGAGGAGAAGGC
m-NLRP3-R	ACGTGTCATTCCACTCTGGC
h-NLRP3-F	TCTGTGTGGACCGAAGCCTAA
h-NLRP3-R	TCCACATGGTCTGCCTTCTC
IL-18-F	TCTTCATTGACCAAGGAAATCGG
IL-18-R	TCCGGGGTGCATTATCTCTAC
IL-1β-F	ATGATGGCTTATTACAGTGGCAA
IL-1β-R	GTCGGAGATTCGTAGCTGGA
Caspase-1-forward	TTTCCGCAAGGTTCGATTTTCA
Caspase-1-reverse	GGCATCTGCGCTCTACCATC

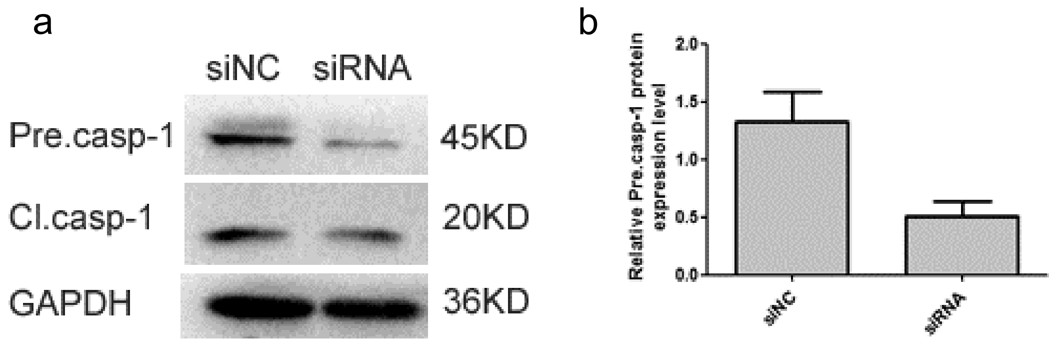

Figure S1. siRNA reduced the expression of caspase-1. a. HeLa cells cultured in 6-well plate were transfected with siRNA of the precursor of caspase-1 for 24 h. Cellular protein was extracted and subjected to Western blot analysis. b. The level of the caspase-1precursor was calculated as fold change relative to GAPDH. Pre. Casp-1: precursor of caspase-1. Cl.casp-1: cleaved caspase-1. siNC: scramble siRNA. Experiment was repeated three times. Representative results were presented.

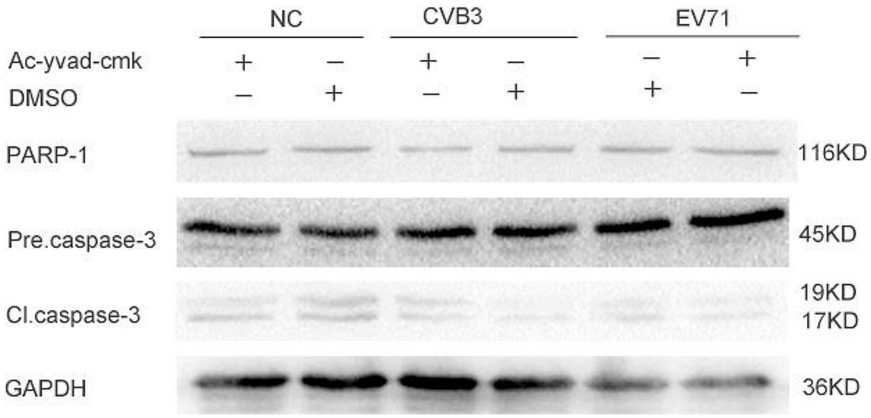

Figure S2. Caspase-1 inhibitor AC-YVAD-CMK shows no impact on apoptosis. HeLa cells were infected with CVB3 or EV71 at MOI = 0.1 and treated with caspase-1 inhibitor Ac-YVAD-CMK at 100 μM for 24 h. Caspase-3 and PARP-1 were analyzed by Western blotting.

Raw materials for Western blots

Figure 1

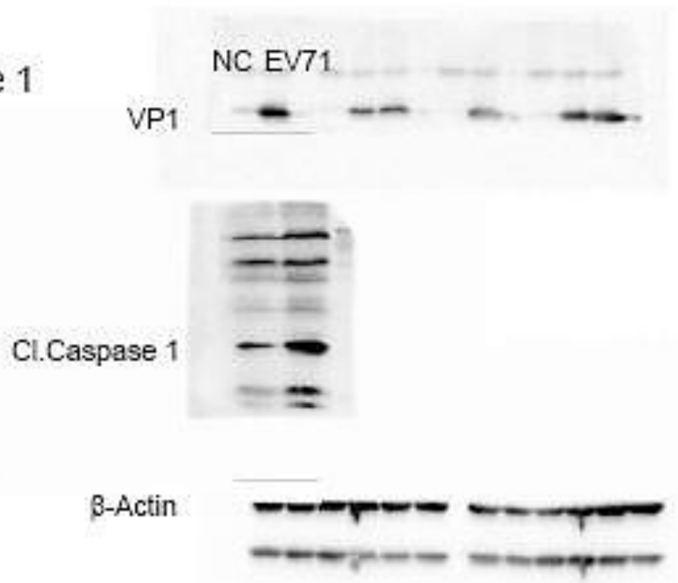

Figure 2 c, d

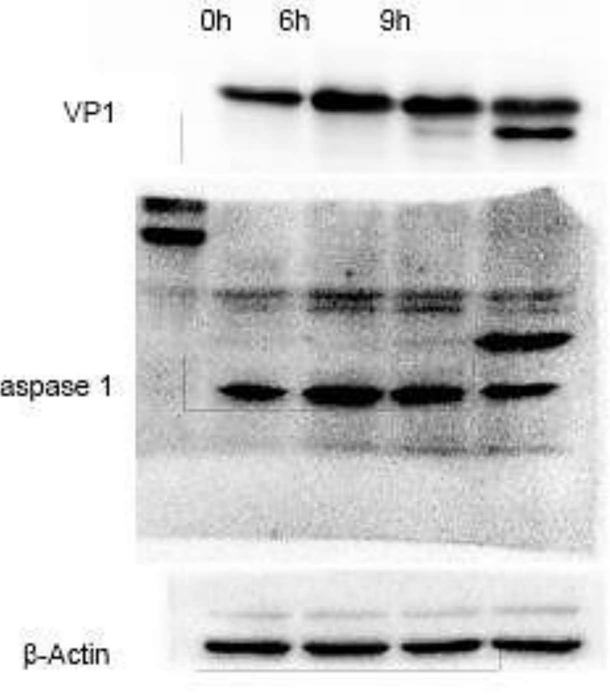

Figure 2 g,h

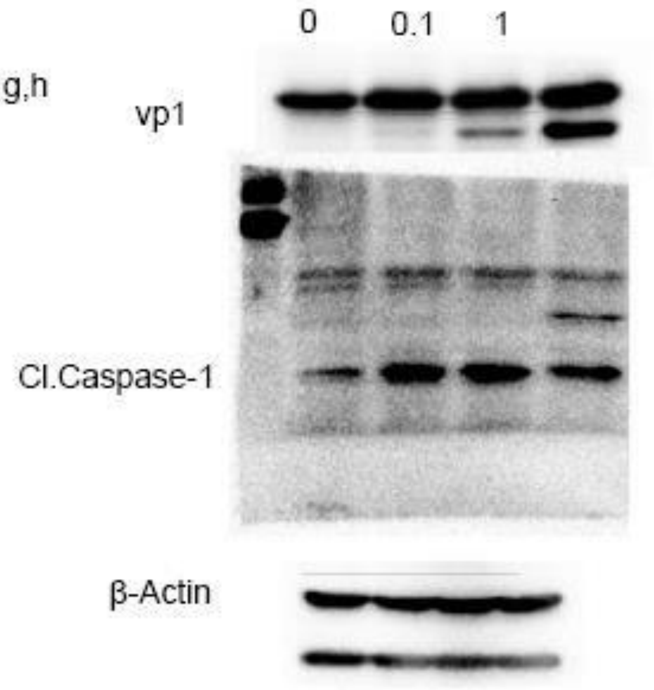

Figure 4

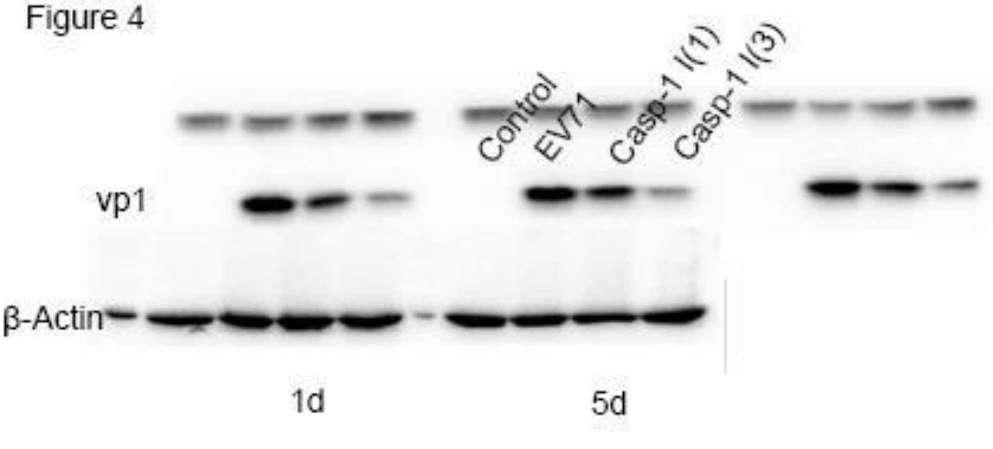

Figure 5

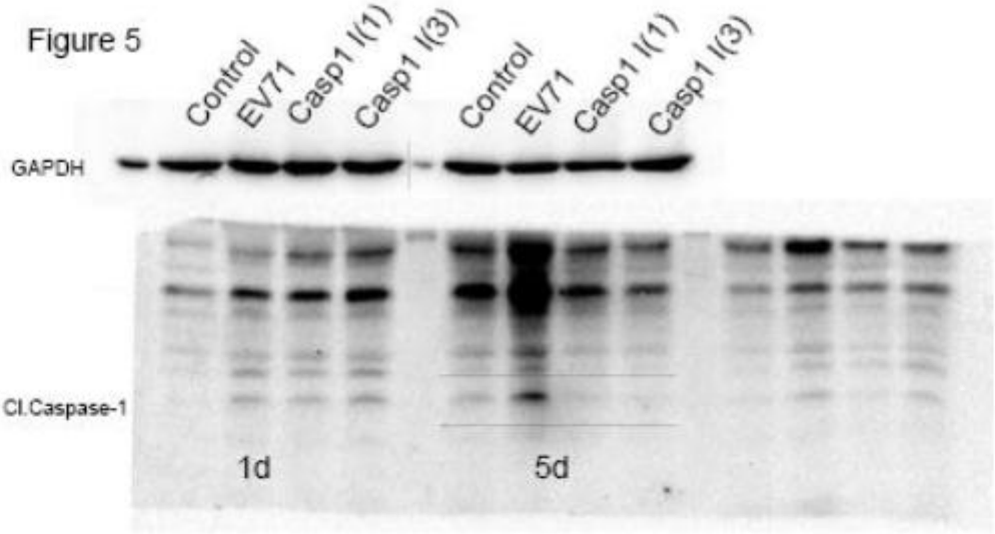

Figure 6 b

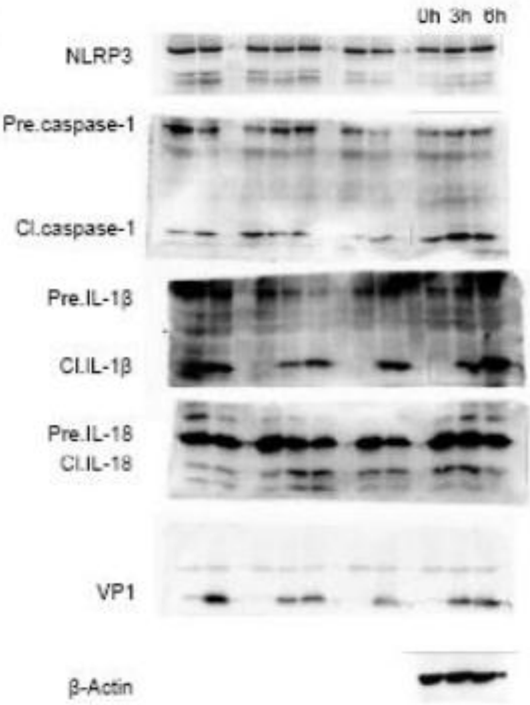

Fig 6 C

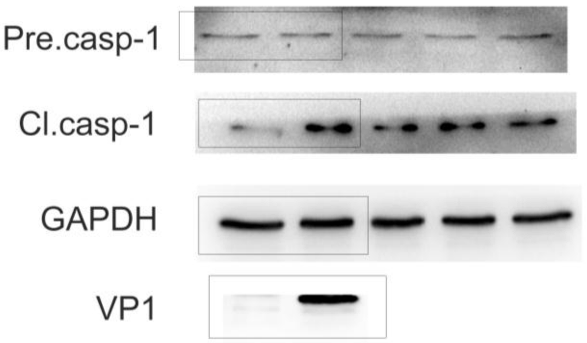

Figure 7

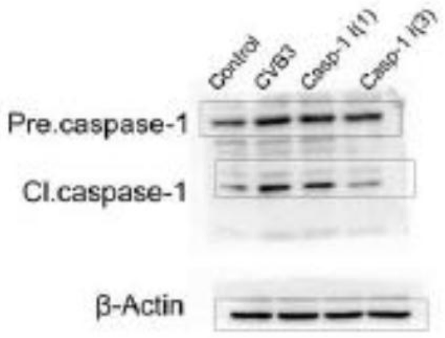

Fig 8 Fig 9

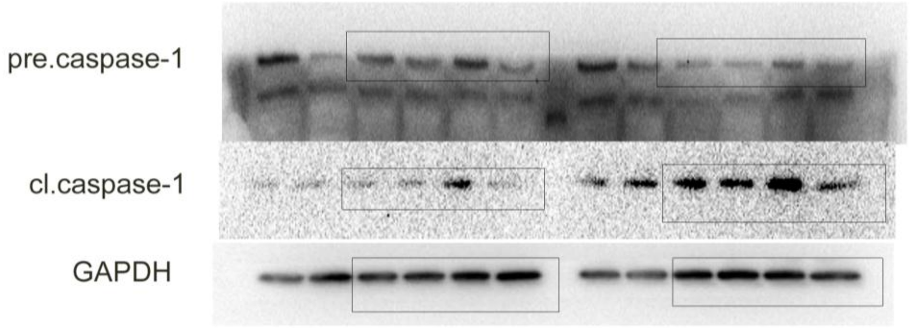

Fig 8

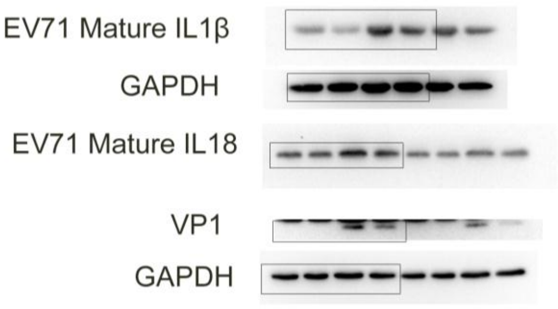

Fig 9

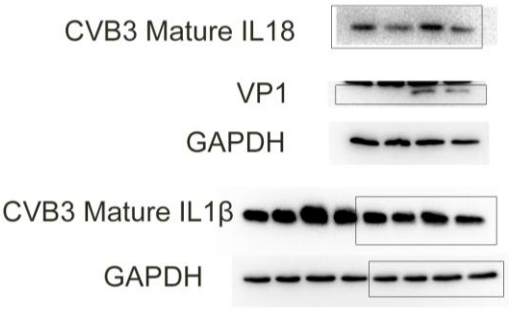

Fig S1

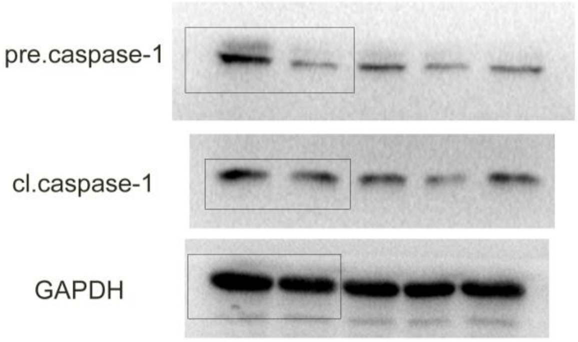
pre.caspase-1
cl.caspase-1
GAPDH

Fig S2

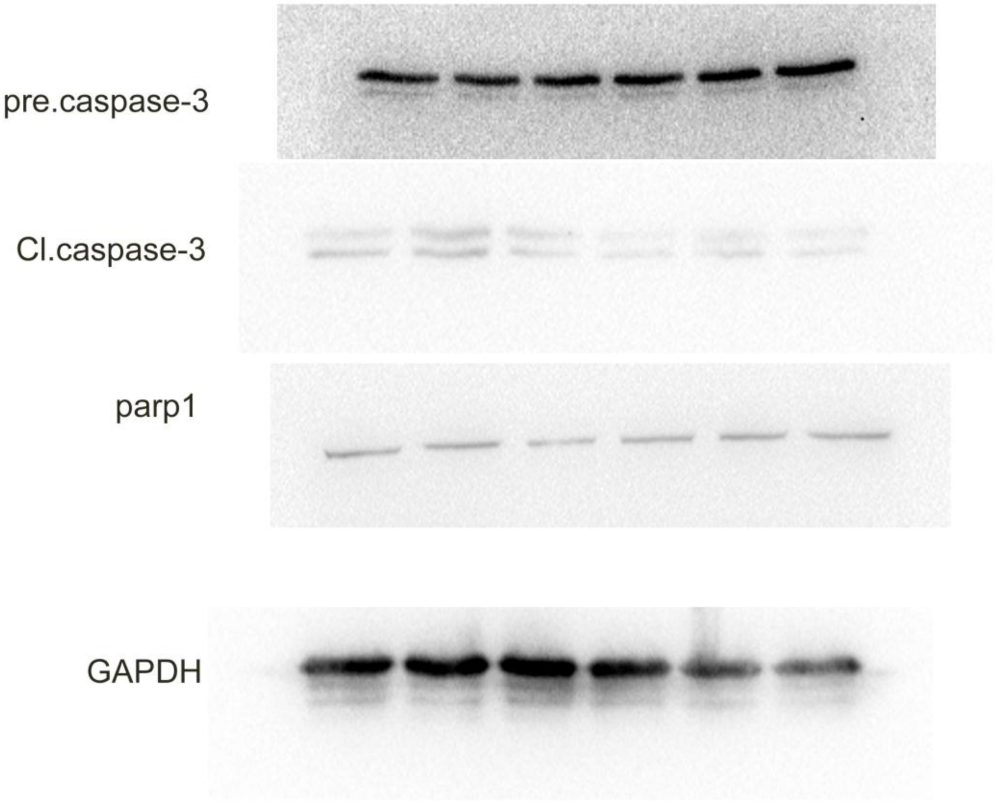
pre.caspase-3
Cl.caspase-3
parp1
GAPDH
